# New Appliances and Things Medical

**Published:** 1904-01-09

**Authors:** 


### NEW APPLIANCES AND THINGS MEDICAL.

### THE "REPELLO" CLINICAL THERMOMETER.

(G. H. Zeal, Thermometer Manufacturer, 82 Turnmill Street, London, E.).

That the shaking down of clinical thermometers has been the cause of many breakages is certain, so that the device which we have before us for obviating such an occurrence may be acceptable. There is at the top of the thermometer a flattened bulb containing mercury, and between this and the registering column of mercury at the other end a small amount of air intervenes. After the temperature of the patient has been taken, pressure on the flat bulb between the finger and thumb drives the contained mercury downwards, forcing the air along, which in turn drives the registering column to below the normal. On the pressure being relieved, the mercury is sucked back again into the flattened bulb and the instrument is once more ready for use. The "Repello" thermometer is compact and excellently constructed, and its practical advantages are likely to lead to its wide adoption.

### "RUBSTITUTE" SURGICAL SOAP.

(Kay Brothers, Limited, St. Petersgate Mills, Stockport.)

We have examined this soap and given it practical trial. Its detergent properties are certainly greater than that found in ordinary soaps, and this is due to the presence of special grease-removing constituents, and not to any excess of alkali or gritty material. We find that sebaceous matter is most efficiently removed from the hands by its use, a property which renders it essentially acceptable to medical men, for whom it is expressly intended. As a preliminary to antiseptic applications, this soap should prove of value in surgical practice.

---

An unfortunate mistake occurred in our report of Veronal in last week's issue. We mentioned 5 grammes as being the dose. The correct dose is ·5 grammes.

